# Angiofluorographic Characteristics of Choroidal Neovascularization associated with Pathologic Myopia

**DOI:** 10.22336/rjo.2020.57

**Published:** 2020

**Authors:** Anca Tomi, Irina Ştefan

**Affiliations:** * Emergency Eye Hospital, Bucharest, Romania; ** Department of Ophthalmology, “Carol Davila” University of Medicine and Pharmacy, Bucharest, Romania; *** Focus Optic Clinic, Bucharest, Romania

**Keywords:** high myopia, choroidal neovascularization, fluorescein angiography

## Abstract

**Purpose:** To evaluate the incidence of choroidal neovascularization (CNV) in high myopic patients who experienced recent visual loss and to describe the characteristics of CNV that develop as a complication of pathologic myopia and were detected by fluorescein angiography (FA).

**Material and methods:** The fluorescein angiograms of 89 patients with high myopia, who registered a recent decrease of visual acuity were reviewed. The incidence of CNV was evaluated and its angiofluorographic appearance described.

**Results:** 172 eyes of 89 patients (six eyes were excluded for different reasons: anophthalmia, dense cataract, history of retinal detachment surgery) were analyzed. Among 89 patients (63 females, 26 males), in 58 cases (~65%), the angiofluorography revealed the presence of a neovascular membrane, and 8 cases presented bilateral CNV. That made a total of 66 eyes. The majority of CNVs were classic (56%), 20% had an occult pattern and 24% were cicatricial. The location was predominantly subfoveal.

**Conclusions:** Considering that the main cause for visual loss in myopic patients is the new-onset myopic CNV, fluorescein angiography should be performed in cases with recent visual loss. When CNV is suspected, FA can demonstrate the presence of myopic CNV, bringing information regarding the type, area, and activity of the CNV and helping exclude other disorders. Due to its location, predominantly sub- or juxtafoveal and to the extended retinal lesions associated with pathologic myopia, the visual prognosis of these patients is poor despite new active treatments.

## Introduction

Although the definition of high myopia or pathologic (degenerative, malignant) myopia is not uniform [**1**], these terms refer to eyes with a refractive error higher than -6 D and an axial length of more than 25,5-26 mm. These eyes develop retinal degenerative changes and complications (biomechanical, neovascular and degenerative) in the posterior pole and/ or in the fundus periphery [**2**,**3**]. These changes give the eye fundus a characteristic aspect (myopic fundus). The myopic maculopathy is the cause for the loss of the best-corrected visual acuity of these patients and was also used to define pathologic myopia (PM) [**4**].

The exact nature of the relation between the level of severity of refractive error associated with specific pathology is still unknown. There is no clearly stated correlation between the severity of refractive error and the specific myopic pathology. Although myopic CNV was reported to occur in eyes without typical myopic degenerative fundus changes and even at lower degrees of myopia, the natural course of the myopic maculopathy was described to follow a progressive pattern [**5**]. The findings range from the early appearance of a tessellated fundus to the development of diffuse atrophy and lacquer cracks, followed by progression to patchy atrophy. The areas of patchy atrophy have the tendency to enlarge and fuse. CNV usually develops adjacent to the area of patchy atrophy or lacquer cracks [**5**,**6**].

Based on long-term clinical observations and considering the progression pattern of the macular changes, Ohno-Matsui et al. formulated a standardized, reproducible classification system for myopic maculopathy defining 5 categories: “no myopic retinal lesions” (category 0), “tessellated fundus only” (category 1), “diffuse chorioretinal atrophy” (category 2), “patchy chorioretinal atrophy” (category 3), and “macular atrophy” (category 4). Three additional features were added to these categories and were included as “plus signs:” (1) lacquer cracks, (2) myopic CNV, and (3) Fuchs spot. PM is defined as myopic maculopathy category 2 or above, or the presence of staphyloma [**4**,**7**]. Recently, a new classification system was proposed by Ruiz-Medrano et al., based on three key factors: atrophy, traction, and neovascularization [**8**].

The fusion of patchy atrophy, the development of CNV, and macular atrophy are the main causes for significant visual loss in myopic patients. 

The choroidal neovascularization (CNV) was reported to occur in 5-10% of the eyes with high myopia [**1**-**4**]. In patients younger than 50 years old, myopia is the main cause for CNV, accounting for more than 60% of the cases [**2**]. The development of the myopic CNV is accompanied by the following acute symptoms: decrease in visual acuity, metamorphopsia and central or paracentral scotoma. On clinical examination, a greyish, flat, small lesion is noticed, usually situated sub- or parafoveally, with or without hemorrhage. Typically, the subretinal membrane is less than 1 disc diameter in size and is located between the neurosensory retina and retinal pigment epithelium (RPE) (type 2), whereas the CNV secondary to age related macular degeneration (AMD) is usually in the sub-RPE space (type 1) [**2**,**4**].

Ohno-Matsui et al. studied the incidence and predisposing findings for choroidal neovascularization (CNV) in a large series of highly myopic patients. They concluded that 1/ 10 of highly myopic eyes with myopic fundus changes in the macula can develop myopic CNV within 3 years and that 30% of these patients will develop CNV in the second eye within the next 8 years after CNV development in the first eye. The risk of developing myopic CNV was higher in eyes with patchy atrophy or lacquer cracks around the macula; during the follow-up period, these patients developed myopic CNV in 20% and, respectively, about 30% of the cases [**9**].

The natural course of CNV is towards regression and scarring, resulting a fibrous pigmented scar (Fuchs’ spot). Chorioretinal atrophy usually develops around the regressed CNV, accounting for the extremely poor long-term visual outcome of the myopic CNV. Yoshida et al. showed that the visual acuity of almost all of the patients dropped to 20/ 200 or less within 5 to 10 years after the onset of CNV [**10**]. Chorioretinal atrophy developing around myopic CNV is the main cause of long-term visual decrease, being influenced by CNV size and patients age [**11**,**12**].

The prognostic of CNV associated with myopia without treatment is not very favorable. Factors generally associated with poor visual prognosis are subfoveal localization, large CNV, lower baseline best-corrected visual acuity and age over 40 [**5**,**13**-**16**]. The progression pattern of the myopic maculopathy was demonstrated in long-term clinical studies [**17**,**18**] and the gradual visual impairment was shown to be caused by the development of CNV or CNV-related macular atrophy and enlargement of macular atrophy. Although anti-VEGF-treatment seemed to be effective in improving and maintaining visual acuity in patients with myopic CNV at 1-year-follow-up [**19**,**20**], the visual acuity gain decreases in most cases in time and deteriorates irreversibly due to advanced myopic chorioretinal atrophy [**21**-**23**].

Sudden vision loss in a high myopia could be due to development of CNV, but these patients have often already poor visual acuity due to posterior staphyloma or chorioretinal atrophy. In the presence of large chorioretinal atrophy and other findings that are common in myopic maculopathy (pigmentary changes, visibility of large choroidal vessels, scarring), the clinical examination may not be able to reveal an obvious CNV in a high myopia which presents with sudden loss of vision [**24**].

It is very important to diagnose a CNV, as the visual deterioration can be influenced by anti VEGF treatment [**1**,**3**,**7**].

Vision loss in a myopic patient can be due to several pathologies and complications of high myopia, including myopic traction maculopathy, epiretinal membrane, vitreomacular traction and myopic full-thickness or lamellar macular hole, retinal hemorrhage due to new lacquer crack formation and macular exudative changes associated with a dome-shaped macula or a staphyloma [**8**]. These diagnoses should be identified, as they require different therapeutic approaches from CNV [**5**].

Fluorescein angiography (FA), indocyanine green angiography, spectral domain optical coherence tomography (SD-OCT), and optical coherence tomography angiography (OCTA) are available diagnostic tools, helpful in making a correct diagnosis [**6**,**24**-**26**]. Different studies evaluated the contribution of FFA and SD-OCT in diagnosis of myopic CNV, finding that FFA has better repeatability and reproducibility to make the diagnosis of myopic CNV compared with SD-OCT. Some concluded that SD-OCT is a better tool to rule out the presence of myopic CNV [**24**]. OCT and now OCTA are non-invasive, easy repeatable imaging tools that aid in an early diagnosis and are important for follow-up of such eyes. OCT alone cannot differentiate myopic CNV from subretinal bleeding due to new lacquer crack formation, which could lead to unnecessary treatment for subretinal bleeding without CNV by anti-VEGF therapy [**5**]. Leveziel et al. found that exudative features of myopic CNV are more obvious on FA than on SD OCT, suggesting that fluorescein angiography should be performed when new-onset myopic CNV is suspected [**25**].

## Materials and Methods

In a retrospective study, we analyzed 89 fluorescein angiograms in myopic patients with different degenerative chorioretinal changes, who presented to the Emergency Eye Hospital Bucharest and to “Retina” Eye Clinic, from January 2005 to December 2014, reporting a recent decrease in visual acuity. Twelve eyes were excluded for different reasons (anophthalmia, dense cataract, history of vitreoretinal surgery, non-myopic eyes); the angiograms of the remaining 166 eyes were reviewed. Inclusion criteria were eyes with high myopia (-6D or more) and recent vision loss (suspicion of presence of CNV). The patient’s history of presenting complaints and best-corrected visual acuity were noted, dilated fundus examination with slit lamp biomicroscopy, color fundus photography and FA were performed.

The presence of exudative activity (leakage) - hyperfluorescence in the early phase with increase in intensity and size in the late phase-, sometimes masked by hypofluorescence due to hemorrhage, the presence of a membrane with minimal or no exudative features but late staining, were attributed to CNV; classic (partial), occult or cicatricial aspects were described and localized. Associated angiofluorographic findings (pigmentary changes, peripapillary atrophy, subretinal hemorrhage, lacquer cracks, patchy chorioretinal atrophy) were documented.

**Fig. 1:**
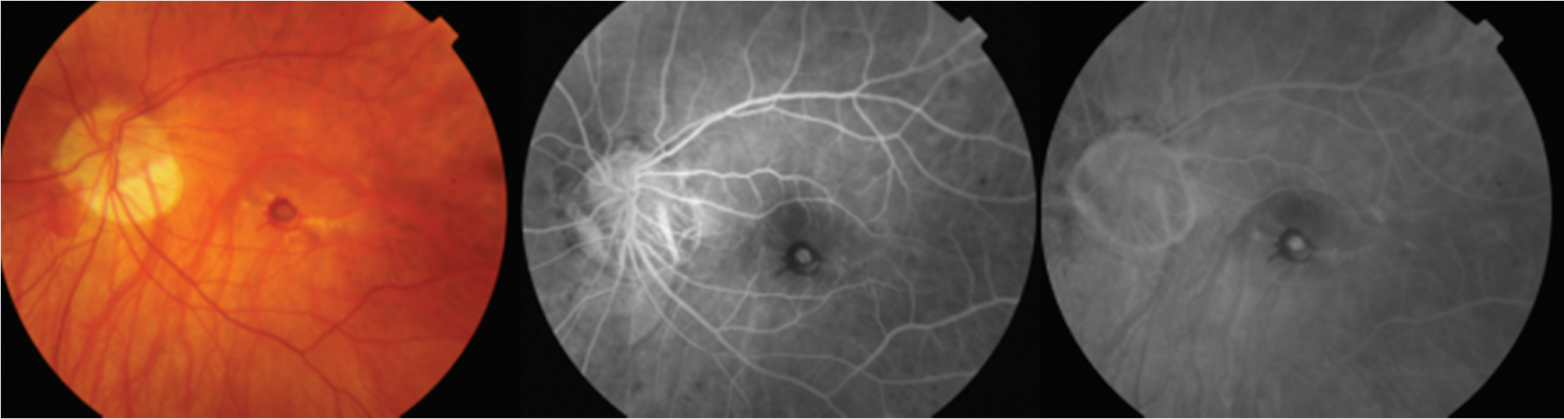
Classic myopic CNV - fundus photography (left) and FA images. The left eye of a 62-year-old male patient presenting a well-defined hyperfluorescence in the early phase (middle), with a hypofluorescent halo, small in size (less than 1/ 2 optic disc diameter), situated subfoveal, with minor to moderate exudation in the late phases (right)

In most of the cases, the angiogram revealed a classic, well defined CNV (well-defined hyperfluorescence in the early phase), sometimes with a hypofluorescent halo, small in size (less than 1/ 2 optic disc diameter), situated sub- or juxtafoveal, presenting minor to moderate exudation in the late phases (**Fig. 1**). The subtle, low exudation of the myopic CNV could be explained by the thinner choroid, characteristic of the high myopic eyes [**8**].

**Fig. 2:**
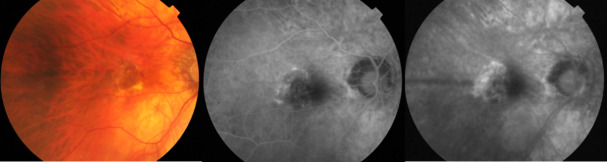
Myopic CNV with occult aspect - fundus photography (left) and FA images. The right eye of a 72-year-old patient presenting a diffuse hyperfluorescence in the early phase (middle), which increases in the late phases (right)

It is rare for choroidal neovascular membranes in myopic eyes to have a poorly defined aspect, but these findings have been described (**Fig. 2**).

**Fig. 3:**
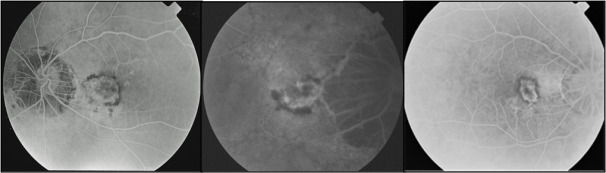
Larger CNV, resembling the subretinal membranes associated with age-related macular degeneration. Early phases FA images from eyes of older patients (59-66 years old)

Larger CNVs can occur especially in older patients (≥ 55 years) (**Fig. 3**). This aspect of CNV occurring in older patients, comparative to those seen in younger patients, may suggest an overlap between myopic CNV and age-related macular degeneration [**25**].

**Fig. 4:**
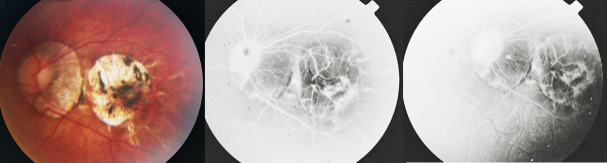
The left eye of a 24-year-old female patient, revealing a cicatricial CNV surrounded by chorioretinal atrophy - fundus photography (left) and FA images (middle and left)

The natural course of CNV is towards regression and scarring, resulting in a fibrous pigmented scar (Fuchs’ spot). The aspect of cicatricial CNV on the angiogram presents hyperfluorescence due to staining, but no exudation. Following regression of the CNV, chorioretinal atrophy forms around the scar, being responsible for the poor long-term visual outcome (**Fig. 4**).

It is often mentioned that CNV in myopic eyes is associated with chorioretinal atrophy or with ruptures of Bruch’s membrane (lacquer cracks), these being the most common predisposing findings for the development of CNV [**9**,**25**].

**Fig. 5:**
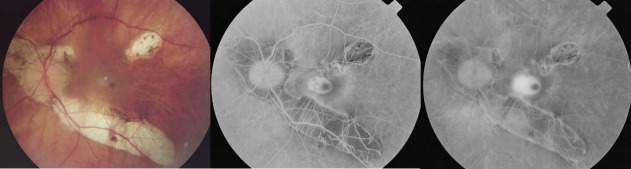
The fundus photography of the left eye of a 51-year-old female patient (left) depicting well defined yellow-white areas of patchy chorioretinal atrophy, with constant aspect throughout the FA (middle and right) with increased visibility of the choroidal vasculature; a CNV is visible (arrows) between the areas of patchy chorioretinal atrophy

Chorioretinal atrophy is of two types - diffuse atrophy and patchy atrophy. Diffuse atrophy appears as yellowish-white areas of atrophy with ill-defined borders. The patchy chorioretinal atrophy appears initially as well defined yellow-white areas situated in the posterior pole and they expand over time, with a tendency to confluate. On the angiogram, these areas appear as well defined, demonstrating increased visibility of the choroidal vasculature, with a constant aspect throughout the FA. CNV can develop on their edge or between the areas of chorioretinal atrophy (**Fig. 5**).

**Fig. 6:**
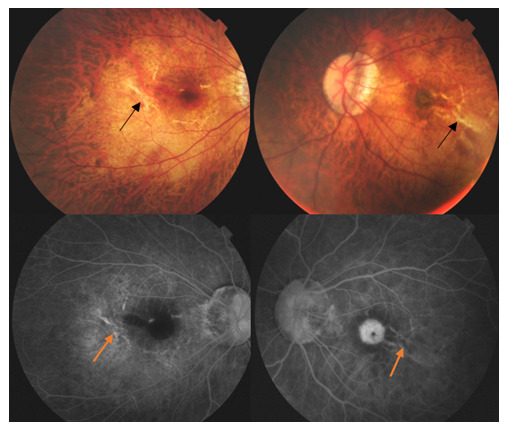
Fundus photographies (upper right and left) and FA (bottom right and left) of both eyes of a 22-year-old female patient reveal the presence of lacquer cracks (arrows). The images of right eye (bottom left) show a subretinal hemorrhage (blocked choroidal fluorescence) without evidence of CNV; the well-defined hyperfluorescence in the left eye (bottom right) demonstrates the presence of a CNV

The ruptures of the RPE-Bruch membrane and choriocapillaris complex (lacquer cracks) appear in the posterior pole as fine yellow radial lesions, hyperfluorescent on the angiogram. Lacquer cracks are usually a sign for the progression of the myopic maculopathy and predispose high myopes to visual loss [**2**,**10**]. They are sometimes associated with small hemorrhages or are predisposing lesions for CNV formation (~30% of eyes with lacquer cracks develop myopic CNV during the follow-up period) [**9**]. A subretinal bleeding is not always associated with choroidal neovascularization in pathologic myopia and can be a sign of new lacquer crack formation (**Fig. 6**).

## Results

166 eyes of 89 subjects were reviewed, 63 females and 26 males. The mean age of our patients was 52 (range 20-83 years); 63 females with a mean age of 50 years (range 20-80) and 26 males with a mean age of 56 (range 22-83). At presentation, only 37 patients (41,5%) were younger than 50 years.

Among the 89 angiograms performed in high myopic patients, 58 (~65% of the cases) revealed the presence of a neovascular membrane, and 8 cases presented bilateral CNV. That makes a total of 66 eyes with CNV. In this group, with CNV, 42 (72,5%) were females and 16 (27,5%) were males.

Considering the aspect of the membrane, it was classic in 56% of the cases (37 eyes), cicatricial in 24% of the cases (16 eyes) and poorly defined in 20% of the cases (13 eyes). Most of the myopic CNV were small (< 1/ 2 disc diameters); in some cases, a larger CNV was revealed, resembling the subretinal membranes associated with age-related macular degeneration. Indeed, this type of CNV was found in our study in 6 older patients (mean age = 61 years, range 56-69).

Regarding the location of the membrane, it was subfoveal in 68% of the cases (45 eyes), juxtafoveal in 23% (15 eyes) and extrafoveal in 9% (6 eyes).

Other angiographic findings documented in the 166 examined eyes, were:

1. pigmentary changes (68 eyes) 40%;

2. peripapillary atrophy (143 eyes) 86%;

3. subretinal hemorrhage (10 eyes, in six eyes associated with CNV) 6%;

4. lacquer cracks in 22 cases 13%;

5. patchy chorioretinal atrophy in 45 cases 27%.

Among the 166 analyzed eyes, 26% of the eyes with lacquer cracks (7 eyes) and 55% of the eyes with patches of chorioretinal atrophy (26 eyes) also demonstrated the presence of CNV on FA. Considering the predisposing factors, the myopic CNV was associated in 10% of the cases (7 eyes) with lacquer cracks and in 39% (26 eyes) with patchy chorioretinal atrophy. In the cases with subretinal bleeding, evidence of CNV could be found in 60% of the eyes (six out of ten eyes); the rest of the hemorrhages were due to lacquer crack formation.

## Discussion

CNV represents a major complication of pathologic myopia, having an important impact on visual acuity. In our series, among 89 patients complaining about recent visual decrease, FA detected the presence of CNV in 58 cases (65%).

It seems that women are more often affected, epidemiologic studies suggesting that myopic CNV is more common in females [**27**]. Our study confirmed this observation (71% of the cases were females). This may confirm the hypothesis that estrogen may play a role in the mechanism of development of CNV [**2**].

Unlike the CNV in age-related macular degeneration, more than 50% of affected myopic patients had a presenting age of 50 or less in most studies [**2**]. In our group, the mean age was 52 with only 37 (41,5%) patients under 50 years of age, suggesting that the visual threatening complications of the myopic maculopathy might develop at an older age. Several studies confirmed that aging is associated with more advanced forms of myopic maculopathy and worse BCVA [**18**,**28**].

Reviewed articles state that approximately 30% of the myopic patients with CNV will develop CNV in the second eye within 8 years [**9**]. In our study, 14% of the cases were bilateral at presentation (8 among 58 patients).

The majority of myopic neovascular membranes revealed a classic aspect (56%). The predominantly subfoveal location (68%) is responsible for the poor long-term prognosis (although the initial response to anti-VEGF therapy is favorable).

Vision loss in highly myopic patients is not always due to CNV, and FA and OCT should be performed to establish a correct diagnosis. In our group of patients, in 35% of the cases, the recent visual deterioration was not due to the development of a myopic CNV; other causes should be kept in mind (for example, lacquer crack formation or subretinal bleeding, or even enlargement of the patches of chorioretinal atrophy).

Although predisposing lesions for CNV (lacquer cracks, chorioretinal atrophy, RPE changes) are well known and easily detected, no prophylactic treatment can be applied [**6**]. Long-term visual outcome of myopic CNV is poor, despite new active treatments [**1**,**5**]. Future therapies should focus on preventing subretinal fibrosis, a characteristic feature of the scarring stage in myopic CNV, which is responsible for the irreversible visual loss [**8**].

## Conclusion

FA enables the understanding of the physio-pathological process in eyes with myopic changes and is useful in detecting the presence and type of myopic CNV, it provides information about its extent, location, and activity, and helps exclude other disorders. It gives an extensive description of the myopic fundus and should be performed as baseline diagnostic test in myopic patients with recent visual decrease. In high myopic eyes, the angiofluorographic detection of a CNV is sometimes difficult due to the multitude of associated lesions and its “low-exudative” characteristics. The information provided by FA should be completed with an OCT imaging. Imaging in high myopic eyes is challenging, as each imaging modality has its limitations; a multimodal approach is important for image interpretation, accurate diagnosis and progression of the disease monitoring. A correct assessment of all fundus changes is necessary before any surgical intervention, whether refractive or cataract surgery, in order to evaluate the visual prognosis.

**Conflict of Interest**

The authors state no conflict of interest.

**Informed Consent**

Informed consent has been obtained from all individuals included in this study.

**Authorization for the use of human subjects**

The research related to human use complies with all the relevant national regulations, institutional policies, is in accordance with the tenets of the Helsinki Declaration, and has been approved by the Ethics Committee of the Department of Ophthalmology, “Carol Davila” University of Medicine and Pharmacy, Bucharest.

**Acknowledgements**

None.

**Sources of Funding**

None.

**Disclosures**

None.
